# Le neurofibrome plexiforme diffus de la cuisse gauche chez une patiente âgée de 78 ans en milieu dermatologique à Bamako

**DOI:** 10.11604/pamj.2017.26.82.11539

**Published:** 2017-02-21

**Authors:** Békaye Traoré, Youssouf Fofana

**Affiliations:** 1 Service de Dermatologie du Centre National d’Appui à la Lutte Contre la Maladie (CNAM), Mali

**Keywords:** Neurofibromes cutanés, neurofibrome plexiforme diffus, taches café au lait, Cutaneous neurofibromas, diffuse plexiform neurofibroma, coffee spots

## Image en médecine

La neurofibromatose type I (NF) ou maladie de Von Recklinghausen est une génodermatose autosomique dominante se manifestant par des anomalies de la peau, du système nerveux, des os et des glandes endocrines. Les neurofibromes plexiformes diffus caractéristiques de cette maladie sont souvent congénitaux, toujours visibles avant cinq ans et tendent à se développer à partir de l’adolescence. Ils sont retrouvés chez 20 à 25% des malades et sont localisés dans 97% des cas à la paupière supérieure. Nous rapportons le cas d’une patiente âgée de 78 ans, présentant depuis l’âge de 8 ans une tuméfaction indolore, molle, avec comme zone d’insertion la lisière fesse gauche-cuisse gauche et se terminait au tiers inferieur de la cuisse gauche. Elle mesurant 25 cm sur sa longueur, 5 cm à sa base d’implantation et 10 cm à son bord libre. Depuis quelques temps, la patiente signalait une sensation de lourdeur de la cuisse gauche. L’examen clinique a objectivé 10 taches café au lait sur le tronc, de nombreux neurofibromes cutanés sur le tronc et les membres. A noter que son fils présentait des lésions similaires. L’examen ophtalmologique a révélé une cataracte évolutive liée à l’âge. Le reste de l’examen clinique était normal. Nous avions évoqué trois hypothèses diagnostiques : un neurofibrome plexiforme diffus le plus probable, un molluscum pendulum et un liposarcome. Le diagnostic de neurofibrome type I a été retenu devant le neurofibrome plexiforme diffus, les Neurofibromes cutanés, neurofibromes plexiformes diffus, taches café au lait et la présence des lésions similaires chez son fils.

**Figure 1:**
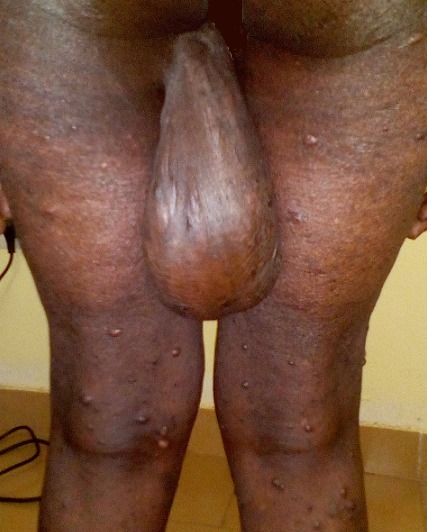
Neurofibrome plexiforme diffus de la cuisse gauche chez une patiente de 78 ans

